# Supplementary material for: Biogeographical Relationships and Diversity in the Peruvian Flora Reported by Hipólito Ruiz and José Pavón: Vegetation, Uses and Anthropology
Source: Biology (Basel). 2023 Feb 13;12(2):294. doi: 10.3390/biology12020294 (PMC9953382; doi:10.3390/biology12020294)
Supplement: Supplementary file 1 [file biology-12-00294-s001.zip › Supplementary Table S2.pdf]

Table S2. Cartographic resources consulted for Peruvian toponymies.

**Colonial cartographic resources consulted**
1735 – Plan scennographique de la cite des rois au Lima capitale du Royaume de Perou. (Map RM. 244185. Available online: https://cartotecadigital.icgc.cat/digital/collection/america/id/602
1750 – Plano de Lima [68]
1750 – Carta de la Provincia de Quito y de sus adjacentes (Map MA00009085). Available online: http://bdh.bne.es/bnesearch/detalle/bdh0000040934
1775–1787 – Maps of the INDIAS HGIS Project. Available online: https://www.hgis-indias.net/ https://dataverse.harvard.edu/dataverse/hgis-indias
1777 – Map of Lima, Peru, as it was before it was destroyed by the earthquake of 1746; Map by Masi, G.T. (David Rumsey Map Collection 14343.050). Available online: https://www.davidrumsey.com/luna/servlet/s/ibq5e1
1788 – Plan del pueblo nombrado San Teodoro de la Colla y de su fuerte San Carlos fabricado en el valle de Vitoc en el... (Map MPD, 13, 015). Available online: http://www.mcu.es/ccbae/es/consulta/registro.do?id=179830
1790 – Plano de la Ciudad de Lima… [69]
1792 – Plano de la Yntendencia de Lima (Map MN 34-A-2). Available online: https://bvpb.mcu.es/es/consulta/registro.do?id=434336
1795 – Plano General de las montañas Orientales al Ryno del Perú : pertenecientes a la Corona de España y confines de... (Map MN 34-A-3). Available online: https://bvpb.mcu.es/es/consulta/registro.do?id=434593
1828 – Plan du Callao de Lima ou Port de Lima. (David Rumsey Map Collection 0233.030). Available online: https://www.davidrumsey.com/luna/servlet/s/zm6l8r
1888 – Mapa del Perú. (Map C-V n 3 (1-10). Available online: https://bvpb.mcu.es/es/consulta/registro.do?id=423426
Stangl, W. *Data: Territorial gazetteer for Spanish America, 1701-1808.* Harvard Dataverse, V2, 2019. https://doi.org/10.7910/DVN/YPEU5E
Stangl, W. *Data: Places gazetteer of Spanish America, 1701-1808.* Harvard Dataverse, V2, 2019. https://doi.org/10.7910/DVN/FUSJD3
Stangl, W. *Data: Mail land routes of Spanish America, 1745-1808.* Harvard Dataverse, V1, 2019. https://doi.org/10.7910/DVN/W4C9H7
Stangl, W. *Basemaps of Spanish American provinces (1701, 1725, 1750, 1775, 1787, 1800, 1808).* Harvard Dataverse, V1, 2020. https://doi.org/10.7910/DVN/RV4LTY
Stangl, W. *Basemaps of Spanish American jurisdictions (1701, 1725, 1750, 1775, 1787, 1800, 1808).* Harvard Dataverse, V1, 2020. https://doi.org/10.7910/DVN/HZJGKA
Stangl, W. *Basemaps of Intendencias (1775, 1787, 1800, 1808).* Harvard Dataverse, V1, 2020. https://doi.org/10.7910/DVN/NBTU2E
Stangl, W. *Basemaps of Spanish American bishoprics (1701, 1725, 1750, 1775, 1787, 1800, 1808).* Harvard Dataverse, V1, 2020. https://doi.org/10.7910/DVN/BY6FYE
Stangl, W. *Basemaps of Audiencias (1701, 1725, 1750, 1775, 1787, 1800, 1808).* Harvard Dataverse, V1, 2020. https://doi.org/10.7910/DVN/PCBLTF
**Post-colonial cartographic resources consulted**
Paz Soldán, M.F. Atlas geográfico del Perú [70]
Sáenz, I.D. Territorio y urbanismo borbónicos [71]
Velarde, H. Itinerarios de Lima [72]
GEO GPS Perú [73]
Instituto Geográfico Nacional [74]
Sistema de Consulta de Centros Poblados [75]
Servicio Nacional de Áreas Naturales Protegidas [76]
